# Utilizing Probiotics for the Prevention and Treatment of Gastrointestinal Diseases

**DOI:** 10.3389/fmicb.2021.689958

**Published:** 2021-08-09

**Authors:** Erin Milner, Benjamin Stevens, Martino An, Victoria Lam, Michael Ainsworth, Preston Dihle, Jocelyn Stearns, Andrew Dombrowski, Daniel Rego, Katharine Segars

**Affiliations:** ^1^Department of Chemistry and Life Science, United States Military Academy, West Point, NY, United States; ^2^Department of Medicine, Uniformed Services University of the Health Sciences, Bethesda, MD, United States

**Keywords:** probiotics, probiotic cultures, probiotic microbiology, food microbiology, probiotic pharmacology, probiotic treatment of gastrointestinal disease

## Abstract

Probiotics are heavily advertised to promote a healthy gastrointestinal tract and boost the immune system. This review article summarizes the history and diversity of probiotics, outlines conventional *in vitro* assays and *in vivo* models, assesses the pharmacologic effects of probiotic and pharmaceutical co-administration, and the broad impact of clinical probiotic utilization for gastrointestinal disease indications.

## Introduction

Probiotic supplement utilization has been steadily increasing based upon the perceived health benefits associated with replenishing the gut microbiome (Saxelin, 2008; Vanderhoof and Young, 2008). A variety of probiotic strains are undergoing clinical trials to treat complex gastrointestinal and inflammatory diseases, but the traditional drug development paradigm associated with preclinical and clinical studies is lacking. In addition, the myriad of probiotic strains and formulations, coupled with a lack of regulatory and quality control associated with commercially available products, has confounded their utilization in patients. Considering these issues, this manuscript focuses on highlighting the history and taxonomy of select probiotics, outlining the data associated with preclinical *in vitro* assays and *in vivo* animal models, and evidence for clinical efficacy and safety for several gastrointestinal disease indications.

## History and Taxonomy of Select Probiotics

### Defining Probiotics

Understanding the complex relationship of microbes within the host gastrointestinal (GI) system has long been an elusive and evolving narrative. While Hippocrates ruminated that “death sits in the bowels,” Nobel Laureate Elie Metchnikoff, who studied immune response, indicated “*lactobacilli* as probiotics (‘probios,’ conducive to life of the host as opposed to antibiotics)” and advocated for the consumption of lactic acid-producing bacteria (Gasbarrini et al., 2016). The World Health Organization (WHO) and the Food and Agriculture Organization (FAO) of the United States adopted a broader definition of probiotic as “live microorganisms which when administered in adequate amounts confer a health benefit on the host organism” (Joint Food and Agriculture Organization World Health Organization Working Group, 2002).

### History of Probiotics

Historical references that date back to 7000 BCE in the Neolithic villages of China and 5000 BCE in Mesopotamia often mention the use of food fermentation techniques (Gasbarrini et al., 2016). Fermentation remained a primary beneficial use of microbes until the late 1800s with the modern concept of the microbiome, which continued to be developed into the early 1900s (Farré-Maduell and Casals-Pascual, 2019). Conducting research at the Pasteur Institute, Metchnikoff studied the benefits of microbes on human health and proposed a theory that, “the dependence of the intestinal microbes on the food makes it possible to adopt measures to modify the flora in our bodies and to replace the harmful microbes by useful microbes” (Metchnikoff, 1907; Gasbarrini et al., 2016). Recognizing health benefits from Bulgarian yogurt and fermented foods, his approach to microbial-treated nutrition revolutionized the dairy industry and promoted a new food industry (Gasbarrini et al., 2016; Farré-Maduell and Casals-Pascual, 2019). A contemporary of Metchnikoff, Dr. Alfred Nissle is credited with identifying *Escherichia coli* strain Nissle 1917 from a soldier who had shown resistance to diarrheal diseases afflicting other soldiers. Dr. Nissle patented the discovery with the trade name “Mutaflor,” a probiotic product that remains currently available (Sonnenborn, 2016; Farré-Maduell and Casals-Pascual, 2019). The probiotic industry continues to flourish due to high consumer demand and the United States market may encompass $77.9 billion by 2025 (Grandview Research, 2019). Probiotics are heavily marketed as part of a preventative healthcare diet, which may appeal to health-oriented consumers. Currently the industry is developing new probiotic supplements such as drops, tablets, and capsules for the rapidly expanding market (Probiotics Market Size Share and Trends Analysis Report By Product, 2019).

### Diversity of Probiotic Strains

The evolving history of human interactions with beneficial microbes has generated a diverse panel of probiotic organisms currently marketed for public consumption. Available probiotics encompass a range of microorganisms as outlined in Table 1, including yeast such as *Saccharomyces* species, as well as bacteria from notably different genera (National Institutes of Health, 2019). A study of over 170 species of *Lactobacillus* concluded that there are significant differences among genomes, phenotypes, and biological effects in experimental models, which leads to variability and inconsistencies when comparing study outcomes (Azaïs-Braesco et al., 2010). Different phenotypic traits (Table 1) exhibited by the diverse organisms may contribute to their utility as probiotic supplements. For example, acid tolerance is likely correlated to probiotic survival as they encounter acidic environments during digestion. *Bifidobacterium animalis* subsp. *lactis* BB-12 is considered to have a high tolerance for acidic conditions and produces bile salt hydrolase enzymes, limiting harm from bile salt exposure in the intestines (Jungersen et al., 2014). Similarly, oxygen tolerance is an important feature of probiotic organisms. Although many gastrointestinal microbes are anaerobic, traditional probiotic bacteria survive in aerobic environments prior to ingestion (Talwalkar et al., 2001). Talwalkar et al. (2001) have reported high oxygen tolerance for several strains of *Lactobacillus acidophilus*, a species commonly used for probiotics. Spore formation may afford an additional benefit for probiotic organisms, supporting their ability to endure sometimes harsh preparation and storage conditions (Cutting, 2011). *Saccharomyces cerevisiae* is a commonly used yeast for fermentation and biofuels (Belda et al., 2019) and *Bacillus subtilis* is a widely studied probiotic species with dormant spores that survive in extreme conditions and a variety of environments (Kovács, 2019).

**TABLE 1:** Selected probiotic products used in the commercial market.

Probiotic (Genus, Species, strain)	Eukaryotic vs Prokaryotic	Gram stain (−/+)	Spore-Forming	Oxygen Tolerance	Formulation	References
*Bacillus coagulans* Nr	Prokaryotic	+	Yes	Aerobic^a^	Capsules	Holt et al., 2000; Sniffen et al., 2018
*Bifidobacterium lactis* (*Animalis*) Dn-173010 (Cncm I-2494)	Prokaryotic	+	No	0.88 (Tolerant via RBGR study)	Yogurt	Holt et al., 2000; Sniffen et al., 2018; Talwalkar et al., 2001
*Bifidobacterium animalis Lactis* Bb-12 (Cncm I-3446)	Prokaryotic	+	No	0.02	Capsules, Powder, Fermented Milk	Holt et al., 2000; Jungersen et al., 2014; Sniffen et al., 2018; Talwalkar et al., 2001
*Escherichia coli* Nissle 1917	Prokaryotic	−	No	Facultative anaerobe^a^	Capsules, Suspension	Holt et al., 2000; Madigan, 2018; Sniffen et al., 2018
*Lactobacillus acidophilus (multiple strains)*	Prokaryotic	+	No	RBGR values ranged from 0.43 to 0.70 among strains tested.	Satchet, Capsules	Holt et al., 2000; Sniffen et al., 2018; Talwalkar et al., 2001
*Lactobacillus casei* Dn-114001 (Cncm I-1518)	Prokaryotic	+	No	0.84 (Tolerant via RBGR study)	Fermented Drink, Yogurt	Holt et al., 2000; Talwalkar et al., 2001
*Lactobacillus rhamnosus* GG (ATCC 53013)	Prokaryotic	+	No	Facultative anaerobe^a^	Yogurt, Capsules	Holt et al., 2000; Sniffen et al., 2018
*Saccharomyces boulardii* Cncm I-745 (ATCC 74012)	Eukaryotic	N/A	No	Facultative anaerobe^a^	Capsules, Sachets	Koutsokali and Valahas, 2020; McCullough et al., 1998; Sniffen et al., 2018; McFarland, 1998; McCullough et al., 1998; McFarland, 1996; Sniffen et al., 2018

*Relative Bacterial Growth Ratio (RBGR) is a quantitative method for assessing oxygen tolerance (Talwalkar et al., 2001). RBGR values are provided in the table for bacteria that were included in the study (Talwalkar et al., 2001). ^a^Not included in the Talwalkar et al. RBGR study.*

## Preclinical *In vitro* Assays and *In vivo* Animal Models

### *In vitro* Antimicrobial Activity

*In vitro* assays have demonstrated several bacterial and yeast species inhibit the growth of pathogenic species (Fijan et al., 2018) or reduce pathogen adhesion to gut epithelial cells (Collado, 2006). In particular, *B. animalis* subsp. *lactis* BB-12 and *Lactobacillus reuteri DSM 17938* inhibited the growth of *E. coli* (Fijan et al., 2018). The concept of employing probiotic species in conjunction with phage treatment to reduce the cytotoxicity of pathogenic *E. coli* was found to be effective at controlling hemorrhagic *E. coli* and ameliorating its cytotoxic effects (Mohsin et al., 2015; Dini et al., 2016). *Lactobacillus paracasei* FJ861111.1 has demonstrated significant inhibition against several common intestinal pathogens including *Shigella dysenteriae*, *E. coli*, and *Candida albicans* via agar diffusion assay models (Deng et al., 2015). A significant decrease in adherence of food-borne pathogens to HT-29 cells (human colon adenocarcinoma cell line) in the presence of *L. paracasei* was also demonstrated (Deng et al., 2015).

*Clostridioides difficile* growth was inhibited in a pH-dependent manner when co-cultured with commercial *Bifidobacterium* and *Lactobacillus* strains (Fredua-Agyeman et al., 2017). The same study also demonstrated inhibition by neutralized cell free supernatant by both strains, although the *Bifidobacterium* strain showed greater inhibition than the *Lactobacillus* strain. In addition, probiotic mixtures have demonstrated effectiveness against *C. difficile* (Deng et al., 2015).

*Listeria monocytogenes*, a common foodborne pathogen, was inhibited by strains of *Lactobacillus plantarum* B7 and *Lactobacillus rhamnosus* D1, demonstrated using spot-on-lawn antagonism (Valente et al., 2019). Probiotic formulations of *L. rhamnosus*, *B. lactis*, and *Bifidobacterium longum* have been shown to reduce proinflammatory cytokines *in vitro* (Sichetti et al., 2018). A Caco-2 cell monolayer *in vitro* assay has been developed to probe the expression of genes involved in the tight junction signaling as a possible mechanism probiotic species utilize to improve intestinal barrier function (Anderson, 2010). Researchers are beginning to elucidate the anti-inflammatory mechanisms associated with *Saccharomyces boulardii* relating to the modulation of protein kinase activity, expression of peroxisome proliferator-activated receptor-gamma, and inhibition of proinflammatory cytokine production (Pothoulakis, 2009). *S. boulardii* has also demonstrated growth inhibition of intestinal pathogens such as *C. albicans, Yersinia enterocolitica, Aeromonas hemolysin, Salmonella Typhimurium* (Ducluzeau and Bensaada, 1982; Altwegg et al., 1995; Zbinden, 1999).

### *In vivo* Animal Models

Several animal models are commonly used for the preliminary assessment of efficacy and safety of probiotics, including mice, zebrafish, and *Drosophila* (fruit fly), which have grown in popularity as cost-effective and simplified models to investigate host-microbiota interactions (Trinder, 2017). While there are limitations associated with preclinical models to study probiotics and host-specific microbiota interactions, these species provide an avenue for investigating the diverse microbiota ecosystem and unraveling the complex interactions prior to costly and logistically burdensome clinical trials.

The differences in gastrointestinal anatomy, physiology, and microbiotas are evident, yet the reduced expense and ease of maintaining zebrafish and *Drosophila* colonies under germ-free (GF) conditions has led to their utilization albeit with limitations (Kamareddine et al., 2020). While human microbiota consists of Actinobacteria, Bacteroidetes, Firmicutes, Proteobacteria, and Verrucomicrobia, *Drosophila* are conventionally populated with Proteobacteria and Firmicutes and zebrafish with Bacteroidetes, Firmicutes, Fusobacteria, and Proteobacteria (Blum, 2013; Xiao, 2015; Kamareddine et al., 2020). In addition to bacterial species, *Drosophila* provide the opportunity to study several yeasts (e.g., Candida and Pichia) (Chandler, 2012; Stamps, 2012). *Drosophila* and Zebrafish models can be employed with conventional microbiota or GF with subsequent selective colonization (Kamareddine et al., 2020). As with all GF models, limitations exist regarding food sources that may contain autoclave-resistant microbial products (Hyun, 1983). While zebrafish are maintained at 28°C in an aquatic environment, which limits the colonization of microbes and confounds the correlation of results to land-based species, they may be colonized by several probiotic bacterial species of interest to humans (to include *Bifidobacterium* and *Lactobacilli*). The simplicity of the zebrafish model allowed researchers to develop an intestinal motility model to assess three strains of peristalsis-promoting probiotics (*Lactobacillus acidophilium*, *L. rhamnosus*, and *B. animalis lactis*) at varying concentrations utilizing a fluorescent dye and image analysis (Lu, 2019) (Wang, 2020). *Drosophila* have been employed as a model to study host-microbiota interactions as a simplified and affordable alternative to mammalian animal models for high-throughput screening of probiotics and to further elucidate host defensive mechanisms against GI pathogens. Zebrafish and *Drosophila* models mimicking gastrointestinal inflammatory conditions have been developed to study host and microbiota interactions and quantify inflammatory biomarker response (Jiang, 2009; Oehlers, 2011; He, 2013).

Murine models have been utilized to study gut microbiota due to their mammalian physiology, but cost is a consideration, especially GF varieties requiring maintenance in special facilities, routine monitoring, and trained personnel. GF mice function as a sterile control or host for selective colonization, but limitations exist based upon their immature intestinal immune system (Laukens et al., 2016). A subset of GF humanized mice has allowed for the replication of a humanized biome with mixed results indicating host-specific interactions that are challenging to replicate (Laukens et al., 2016). Strain, genotype, phenotype, and gender differences further confound the extrapolation of results and have led to the development of guidelines to control murine microbiota model variability (Laukens et al., 2016).

Germ-free mice were utilized to study the involvement of microbiota in gastrointestinal diseases such as inflammatory bowel disease and colitis, and subsequent prophylactic and treatment modalities of probiotics. For example, Lactic acid bacteria (LAB) were investigated to prevent chronic inflammation. *L. plantarum* persisted in the digestive tracts of mice with TNBS-induced colitis for up to ten days after treatment without harmful effects exhibited. Overall, intestinal inflammation decreased and there was no incidence of bacterial dissemination (Pavan et al., 2003; Hu et al., 2019). *L. reuteri* has been shown to reduce *C. difficile* infection in mice. Based on a recent study, a single dose of *L. reuteri* biofilm is efficacious in the prevention of *C. difficile* colitis. When administered either therapeutically or prophylactically, it can reduce the frequency and prevalence of the infection (Shelby et al., 2020). Researchers have also combined conventional and GF mice and zebrafish models to investigate how host-specific interactions modify microbiota communities (Rawls, 2006). Zebrafish were colonized with mouse gut microbiota and mice were colonized with zebrafish microbiota, which allowed for comparison of the host and transplanted communities at the phylogenetic level. Their results indicated the host gut altered the microbiome after transplantation between these species, which further indicates the limitations of extrapolating data across species.

## Regulation, Clinical Efficacy, and Safety

Probiotics in the United States could be regulated by the Food and Drug Administration (FDA) as drugs, biologics, or dietary supplements based on the intended use (U.S. Food and Drug Administration National Institute of Health National Institute of Allergy and Infectious Diseases, 2018; National Institutes of Health National Center for Complementary and Integrative Health, 2021). These products are under the purview of different centers within the FDA, often covered by different laws. As such, it may not always be clear to end-users how a commercially available probiotic is marketed. When considered dietary supplements, probiotics are regulated according to the Dietary Supplement Health and Education Act of 1994 (DSHEA) and the requirements tend to be more in line with food safety expectations rather than drug or biologics (U.S. Food and Drug Administration National Institute of Health National Institute of Allergy and Infectious Diseases, 2018; Venugopalan et al., 2010; U.S. Food and Drug Administration, 2019). A key difference between dietary supplement and drug/biologic regulation lies in the requirements that manufacturers must meet before marketing their products. The FDA typically requires thorough review of *in vitro, in vivo*, and clinical studies before drug approval or biologic licensing, which may be submitted in the form of detailed applications designed to evaluate safety and effectiveness (U.S. Food and Drug Administration, 2014, 2017). As dietary supplements, probiotics are primarily subjected to FDA premarket review only when they are comprised of a “new dietary ingredient,” which DSHEA describes as “dietary ingredient that was not marketed in the United States before October 15, 1994” (National Institutes of Health Office of Dietary Supplements, 1994). For dietary supplements, it is left to the manufacturers discretion to establish whether their ingredient is new (U.S. Food and Drug Administration, 2020), which could potentially cause inconsistencies in which probiotics are reviewed by the FDA. Manufacturers of dietary supplements with new dietary ingredients are expected to submit a premarket notification to the FDA, which differs from drug approval or biologics licensing in the degree of safety/efficacy evaluation and resulting regulatory decision (U.S. Food and Drug Administration National Institute of Health National Institute of Allergy and Infectious Diseases, 2018). If the manufacturer does not deem their dietary ingredient to be “new,” the FDA generally relies on the companies to ensure that their products meet marketing and labeling requirements. Consequently, the same probiotic product could have very different testing requirements and regulatory processes according to how it will be labeled for use.

Once marketed, labeling and health claims are also a potential complicating factor in probiotic usage. According to DSHEA, dietary supplement labeling “may not claim to diagnose, mitigate, treat, cure, or prevent a specific disease or class of diseases (National Institutes of Health Office of Dietary Supplements, 1994).” It is relevant to note that per DSHEA, labeling statements are allowable if “the statement claims a benefit related to a classical nutrient deficiency disease and discloses the prevalence of such disease in the United States, describes the role of a nutrient or dietary ingredient intended to affect the structure or function in humans, characterizes the documented mechanism by which a nutrient or dietary ingredient acts to maintain such structure or function, or describes general well-being from consumption of a nutrient or dietary ingredient…” (National Institutes of Health Office of Dietary Supplements, 1994). This distinction in acceptable labeling could result in ambiguous claims concerning probiotic health benefits, which may not be easily interpreted by the public. Dietary supplement advertising falls under the regulatory purview of the Federal Trade Commission rather than the FDA (U.S. Food and Drug Administration, 2019) and shared federal jurisdiction increases the complexity of monitoring product claims marketed to consumers. Further, the National Institutes of Health has noted reports of probiotics with potentially dangerous contents that did not match the labeling (National Institutes of Health National Center for Complementary and Integrative Health, 2021). The Council for Responsible Nutrition and International Probiotics Association offers labeling guidance to probiotic manufacturers that includes specifying detailed information at the strain level concerning the type, quantity, and storage conditions of the organisms; however, these parameters are presented as recommendations rather than requirements (International Probiotics Association, 2017). Historically, many studies have noted that laboratory testing does not always corroborate the presence of microorganisms claimed in probiotic labeling (Yeung, 2012). More recently, Metras et al. (2021) conducted a study to compare labeling information with the actual microbial content of five commercially available fermented kefir products regulated as dietary supplements. Their results demonstrated inconsistencies between the information claimed on the labeling and the actual species and quantified colonies that were present under the conditions tested (Metras, 2020).

Due to lack of probiotic prescribing information, healthcare providers do not have succinct resources outlining the indications, dosage and administration, contraindications, warnings and precautions, adverse reactions, drug interactions, and use in specific populations (Reid et al., 2019). Consequently, much of the knowledge concerning safety and efficacy is derived from a patchwork of literature, which must be reviewed and interpreted by people interested in clinical applications for probiotics. A more standardized approach to probiotic regulation, testing, and labeling processes would be beneficial to reduce the variability and inconsistency that currently exists in the literature. For example, consistent *in vivo* testing requirements could generate a more robust body of literature concerning whether a given strain is effective against a specific condition and how formulation may affect delivery and disease outcome, information that is generally lacking at present (Sniffen et al., 2018).

### Microbiota and Gastrointestinal Pathology and Pathophysiology

The dynamic mix of host cells and microorganisms have evolved (Bäckhed et al., 2005; Ley, 2006) and integrated into critical physiological functions such as shaping the intestinal epithelium (Natividad and Verdu, 2013), digestion (Chang and Martinez-Guryn, 2019), regulating host immunity (Gensollen et al., 2016), and protecting against pathogens (Bäumler and Sperandio, 2016). The microbiota contributes to carbohydrate, lipid, protein metabolism and digestion (simple sugars, fatty acids, and amino acids) via the principal absorption sites of the major nutrients. The small intestine has two primary functions, digestion and absorption, that are affected by the GI microbiota. Segmental movements of the small intestine mix ingested materials withpancreatic, hepatobiliary, and intestinal secretions along with microbiota enzymes. Metabolomic advances are beginning to elucidate the interwoven relationship between healthy and diseased mucosa-associated microbiota, which are strongly correlated to dietary sources (Eetemadi et al., 2020).

**FIGURE 1:**
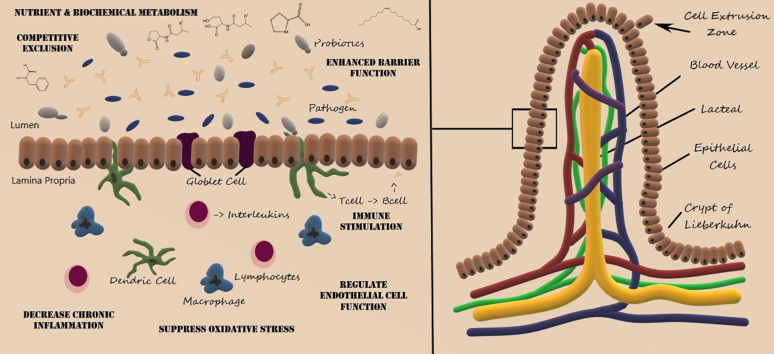
Structure of the villus of the small intestine and representative probiotic mechanisms of action. Artwork by Audrey Milner.

As shown in Figure 1, the villus consists of a central lymph channel (lacteal) surrounded by a network of blood capillaries within lymph tissue bordered by epithelial cells (Noah et al., 2011). Surrounding each villus are small pits called the crypts of Lieberkuhn, which contain undifferentiated cells that proliferate rapidly and migrate toward the tip of the villus and are shed into the intestinal lumen (Noah et al., 2011). Maturation and migration from the crypts to the tip of the villus requires 5–7 days and approximately 20–50 million epithelial cells are extruded into the intestinal lumen each minute (Gehart and Clevers, 2019). The cellular composition within the gastrointestinal tracks was recently estimated at 3 × 10^13^ host cells along with 4 × 10^13^ microbiota cells (Sender et al., 2016), whereby colonization and microbial diversity occurs in parallel with the development of the mucosal absorption and immune system response (Aidy et al., 2013). Both metabolic processes and signal transduction pathways between the host and microbiota are intimately linked and alterations within the gastrointestinal environment can lead to pathophysiological consequences (Bermudez-Brito et al., 2012; Zhang, 2019).

Although not fully elucidated, the enhanced mucosal barrier function, inhibition of pathogen adhesion, and competitive exclusion of pathogenic microorganisms are also mediated by gut microbiota and probiotic administration (Bermudez-Brito et al., 2012; Cornick et al., 2015). The villous epithelium consists of mucus producing goblet cells and absorptive cells, which are responsible for the absorption of nutrients and medications. Pathogenic microbes and microbial toxins can disrupt goblet cell function and disrupt the integrity of the mucus barrier, leading to chronic inflammatory diseases (Cornick et al., 2015). Probiotics, such as *L. rhamnosis* and *L. plantarum*, have been shown to enhance the mucus barrier (Wang et al., 2014), regulate epithelial cell function (Ohland and Macnaughton, 2010), suppress oxidative stress (Ciorba et al., 2012), and mitigate immune response thereby decreasing chronic inflammation (Mann et al., 2013).

#### Effect of Antibiotics on the Gut Microbiota

It is important to acknowledge the complicated relationship between gastrointestinal disease, gut microbiota, probiotics, and antibiotics. Maintaining the appropriate gut microbiota ecosystem in the age of antibiotic treatments (Gibson et al., 2015) and resistance (Schaik, 2015) is of particular importance. Common pathogenic strains that contribute to GI diseases are *Campylobacter*, *C. difficile, E. coli, Helicobacter pylori, Salmonella* species, *Shigella, Staphylococcus aureus, Clostridium perfringens, Listeria monocytogenes, Bacillus cereus*, and *Y. enterocolitica* (Alby and Nachamkin, 2016; Harmon, 2017; Sandra and Tallent, 2020). Symptoms include diarrhea, vomiting, abdominal pain, and heartburn (Alby and Nachamkin, 2016). However, elders and immunocompromised individuals can have serious complications from GI diseases due to potentially weakened immune systems. Oftentimes GI infections are treated with antibiotics, however, the rise of antibiotic resistant bacterial strains is yielding mixed results. Thus, researchers are looking for new alternatives to combat GI infections, for example, the use of probiotics and fecal transplant.

The introduction of antibiotics alters the microbial ecosystem, which can lead to a limited gut microbial diversity (Fjalstad et al., 2018) and the reestablishment of pathogenic infections (Yoon and Yoon, 2018). The necessity to limit antibiotic treatment in neonates is prominent due to potentially disease-promoting microbiota alterations. Antibiotic exposure in infants and young children may have significant impacts on the microbiota during critical periods of development (Silverman et al., 2017). Antibiotic treatments can cause reduced colonization rates and increased risk of multi-drug resistant (MDR) strains (Fjalstad et al., 2018). Conversely, the natural microbiome recovery post antibiotic administration has been explored in murine models (Ng, 2019) and documented in human studies with varied microbiome population effects given the heterogeneity associated with antibiotic treatment regimens (MacPherson, 2018; Elvers, 2020).

#### Antibiotic-Associated Diarrhea

Antibiotic-associated diarrhea (AAD) is a common side effect of antibiotic usage, which affects up to 30% of patients administered antibiotics (McFarland, 2007). There are believed to be several ways by which antibiotics cause diarrhea, including killing beneficial microbes and influencing metabolic processes (McFarland, 1998; Silverman et al., 2017). Some antibiotics, such as amoxicillin-clavulanate, ampicillin, cephalosporin and clindamycin may cause AAD with increased incidence (McFarland, 1998; Silverman et al., 2017). In addition to the type of antibiotics used, individual patient susceptibility may also influence the development of AAD (Barbut and Meynard, 2002).

Probiotics are a common choice for patients suffering from AAD and have been widely advocated as a safe and effective way to reduce adverse side effects of antibiotics on gastrointestinal function (Mills, 2018). *C. difficile* infection can occur following the antibiotic-associated loss of intestinal flora, potentially increasing serious diarrheal disease (Silverman et al., 2017), and is one of the principal causes of AAD (Young et al., 2018). *C. difficile* infection has an incidence of approximately 500,000 infections per year in the United States and approximately 30,000 cases resulting in fatality within 30 days (Mada and Alam, 2020). A meta-analysis of 25 randomized controlled trials indicated probiotics reduced the relative risk of ADD (RR = 0.43, 95% CI 0.31, 0.58, *p* < 0.001) and the analysis of six randomized trials led to statistically significant reduction in *C. difficile* (RR = 0.59, 95% CI 0.41, 0.85, *p* < 0.005) (McFarland, 2006). In particular, *L. rhamnosus* GG *and S. boulardii*, were identified as effective for treating AAD and *S. boulardii* was particularly effective for reducing *C. difficile* infection.

*Lactobacillus rhamnosus* GG and *S. boulardii*, have been proposed to maintain the gut microbiota and production of energy via fermentation as well as competition with pathogen binding sites (Hickson, 2011; Vecchio et al., 2015). However, the mechanisms of action are still unknown (Hickson, 2011). When investigating the efficacy in reducing AAD, *S. boulardii* resulted in a relative risk of 0.47 [95% confidence interval (CI) = 0.35, 0.63; *p* < 0.001] and 0.31 (95% CI = 0.13, 0.72; *p* = 0.006) for *L. rhamnosus* (Hickson, 2011). A recent study demonstrated that *S. boulardii* acts to reduce toxin A-receptor binding by releasing a protease that cleaves toxin A, an exotoxin released by *C. difficile* (Castagliuolo et al., 1996; Pothoulakis, 2009). A controlled clinical trial focusing on the prevention of *C. difficile* infection with *S. boulardii* indicated a reduction of *C. difficile* relapse in the recurrent treatment group of patients receiving high-dose vancomycin (*p* = 0.05), furthering support for usage (Surawicz et al., 2000). However, two studies examining *S. boulardii* found that the probiotic had no significant effect in treating *C. difficile* associated diarrhea (AD; Surawicz et al., 1989; Kotowska et al., 2005). *L. rhamnosus* is reported to increase the production of gut mucin, which functions as a barrier defense for the epithelium thereby reducing the effects of *C. difficile* AD (Mack et al., 1999).

A study investigating 29 probiotics found that Bio-K+ (a probiotic cocktail comprised of *L. acidophilus, Lactobacillus casei, and L. rhamnosus*) survived the GI environment inhibiting growth and toxin neutralization (Auclair et al., 2015). BIO-K+ also decreased the production of methicillin-resistant *S. aureus* by 99%, providing evidence for growth inhibition (Karska-Wysocki et al., 2010). Furthermore, in terms of toxin neutralization, Bio-K+ demonstrated anticytotoxic effects in a toxin neutralization assay that tested 13 strains (Auclair et al., 2015). *B. bifidum* and *Streptococcus thermophilus* were supplemented into an infant’s diet and showed decreased occurrence of diarrheal symptoms (Saavedra et al., 1994). Regarding safety concerns, a report investigated the production of putrescine via *B. bifidum*, but determined the concentrations were consistent with safe food sources (Kim et al., 2018).

#### *Helicobacter pylori* Infection

*Helicobacter pylori* infection (HPI) occurs in roughly 60% of the world’s population and can cause various gastroenterological disorders including conditions associated with dyspepsia, peptic ulcer, and stomach cancers (Chey et al., 2017; Hooi, 2017). Available treatment methods for HPI usually involve combinations of two or three antibiotics with a proton pump inhibitor, referred to as “triple therapy” or “quadruple therapy,” respectively (Ables et al., 2007; Chey et al., 2017). Total eradication is rare, as the efficacy of these treatments tend to vary and are impacted by antibiotic resistant strains (Higuchi et al., 2006; Sun et al., 2010). Studies have shown that using probiotics in conjunction with other treatments may aid in eradication of *H. pylori*. When combined with a triple therapy of omeprazol, clarithromycin, and amoxicillin, pre-treatment of patients with *L. acidophilus, S. faecalis*, and *B. subtilis* for two-weeks improved the eradication rate by 18.7% compared to the control (Du et al., 2012). A 24-month clinical trial involving nearly 500 subjects found similar results with a combination of probiotic treatment and triple therapy increasing eradication rate by 7% (Rieko et al., 2020). It is proposed that this colonization reduction may be due to a decrease in the biotic load despite *H. pylori* antimicrobial resistance (Du et al., 2012). In the afore mentioned studies, research was performed with pretreatment of probiotics, but not with concurrent treatment alongside the triple therapy. Although some studies have shown successful *H. pylori* eradication with additional probiotic treatment, a meta-analysis performed by Lu et al. (2016) suggests that probiotic use provided little benefit over a placebo. Additionally, Cindoruk et al. (2007) reported that using *S. boulardii* along with an antibiotic triple therapy did not result in a statistically significant eradication increase but did reduce symptoms associated with treatment when compared to a placebo (Cindoruk et al., 2007). Overall, additional studies are required to elucidate the effectiveness of probiotics with HPI (Chey et al., 2017) and the effect of antibiotic treatments on the survival of probiotics (Rieko et al., 2020).

#### Blastocystis

*Blastocystis* species are anaerobic intestinal protozoans, typically considered to be pathogenic although there is increasing evidence that they should be considered a commensal (Sinclair, 2016; Deng, 2021). Infections may be present in both asymptomatic and symptomatic individuals, potentially demonstrating generalized gastrointestinal symptoms (Coyle et al., 2012; Wawrzyniak et al., 2013). There are medications available for *Blastocystis* infections, including the commonly used metronidazole or trimethoprimsulfamethoxazole; however, clinical indications for when to treat remain somewhat ambiguous (Coyle et al., 2012; Sekar and Shanthi, 2013; Wawrzyniak et al., 2013). Further, there have been case reports of treatment that did not eradicate *Blastocystis* (Roberts et al., 2014), and emerging resistance to metronidazole has been reported (Sekar and Shanthi, 2013). Despite the apparent need for clarity regarding effective treatment of *Blastocystis* infections, limited clinical data is available investigating probiotics to support treatment of *Blastocystis*. One trial examined the use of *S. boulardii* in lieu of metronidazole in symptomatic children with *Blastocystis hominis* positive stools. After one month, those treated with doses of *S. boulardii* had a 94.4% clinical cure rate and 73.3% clinical cure rate was reported for those who received the standard metronidazole treatment; whereas the parasitological cure rate was similar between each group (Dinleyici et al., 2010). While *S*. *boulardii* efficacy against *B. hominis* has not been thoroughly characterized, it has been suggested that probiotics may displace protozoan pathogens in the gastrointestinal tract and potentially alter the patient’s immune response, thus improving the clinical outcome (Vitetta et al., 2016). An *in vitro* study evaluated the use of some probiotic bacteria and demonstrated that *L. rhamnosus*, *L. lactis*, and *Enterococcus faecium* reduced *Blastocystis* under the culture conditions tested (Lepczyńska and Dzika, 2019). In addition, a similar study which examined the interactions between Blastocystis subtype 7 (ST7) and various gut bacteria, found that *Blastocystis* ST7 reduced beneficial *Bifidobacterium* and *Lactobacillus* species in mice (Yason, 2019).

#### Acute Gastroenteritis

Acute gastroenteritis (AG) refers to inflammation within the gastrointestinal track, most often accompanied by an infection and characterized by sudden emergence of symptoms including nausea, vomiting, watery stool, and abdominal discomfort (Graves, 2013; Hartman et al., 2019). Symptomatic treatment, anti-infective therapy, and addressing dehydration are the primary clinical focus (Zollner-Schwetz and Krause, 2015; Hartman et al., 2019). An estimated 1.5–2.5 million children die each year from infectious gastroenteritis. Molecular, immunoassay and culture methods are utilized to diagnose the diverse bacterial and viral pathogens leading to the etiology (Humphries, 2015; Tarr, 2019). Probiotic products may also be useful for AG, in part by modifying the gastrointestinal microbiome, as well as exerting effects on physiology, such as anti-inflammatory responses and fortification of epithelial cell tight junctions (Kluijfhout et al., 2020). The probiotics *lactobacilli* and *S. boulardii* are the most researched in treating this disease (Kluijfhout et al., 2020). *S. boulardii* produced a significant decrease in diarrhea (14.0% day 1; 13.1% day 2) when administered to treat AG (Kluijfhout et al., 2020). In terms of the mechanism of action, *S. boulardii* has been shown to disrupt the production of proinflammatory cytokines and interfere with inflammation nuclear factors (Sougioultzis et al., 2006). Conversely, another study found that *L. rhamnosus* and *Lactobacillus helveticus* did not demonstrate a decrease in presence or symptoms associated with viral infection (Freedman et al., 2020).

#### Necrotizing Enterocolitis

Necrotizing enterocolitis (NEC) is the leading cause of neonatal morbidity and mortality (Neu and Walker, 2011). Although the etiology of NEC is not clear, immature immune function and alteration of the intestinal microbiome post antibiotic treatment may be contributing factors (Xiong et al., 2020). Common symptoms include feeding intolerance, lethargy, bloating, and bloody stools. Treatment focuses upon fluid replacement, nutrition, anti-infective therapy, and surgery. There have been several meta-analyses indicating probiotics prevent NEC. A meta-analysis of 24 randomized controlled trials demonstrated clinical efficacy (RR 0.65, 95% CI 0.52–0.81; 17 studies, 5,112 infants) in reducing the incidence of NEC utilizing *Lactobacillus* monotherapy or co-administration with *Bifidobacterium* (AlFaleh and Anabrees, 2014), which are present in the microbiomes of healthy infants (Eugenia Bezirtzoglou, 2011). Another meta-analysis (RR 0.36, 95% CI, 0.24–0.53, *n* = 7345 infants) showed prophylactic efficacy of developing NEC in probiotic-treated infants (Chang, 2017). Although, a 2015 study involving 1,315 infants indicated no evidence of the benefit of using *Bifidobacterium breve* BBG-001 for the prevention of NEC in preterm infants, underscoring the species variability relative to clinical outcomes (Costeloe et al., 2016).

#### Irritable Bowel Syndrome and Functional Bowel Disorders

Irritable Bowel Syndrome (IBS) occurs on a spectrum from mild to severe and includes recurrent abdominal discomfort and pain, bloating, and stool alterations varying between constipation and diarrhea (Defrees, 2017). The etiology of IBS remains unclear and the symptoms are often associated with differential diagnoses (Aziz and Simrén, 2021). Studies involving probiotics have shown clinical benefits in treating IBS patients such as fecal consistency, flatulence, bloating, the number of symptoms present, appetite, bowel frequency, and nourishment (Harris and Baffy, 2017). Recent clinical data has supported utilizing probiotics to modify the microflora within the gut to reduce inflammation (Boirivant and Strober, 2007).

In the case of a clinical study conducted to determine the ability for *L. acidophilus* and *B. animalis* subsp. *lactis* to treat bowel disorders, the difference between the test and placebo groups were not statistically significant for the primary endpoints of GI relief and satisfaction. However, several of the symptoms studied significantly improved when compared to the control group. Abdominal bloat showed statistically significant improvement when compared to the control group with a *p*-value of 0.009 after 4 weeks and a value of 0.06 after 8 weeks (Ringel-Kulka et al., 2015). Additionally, there were no significant changes in standard blood test ranges and fecal samples as safety indicators (Ringel-Kulka et al., 2015).

*Lactobacillus acidophilus* CL1285, *L. casei* LBC80R and *L. rhamnosus* CLR2 have been identified as potential treatments to relieve the symptoms associated with IBS (Preston et al., 2018). This combination of probiotics demonstrated endpoint improvement including abdominal pain, days in pain, buildup of gas within the stomach, and stool habits. Primarily mild to moderate safety concerns were reported in some treatment and placebo participants; however, the authors concluded the concerns could not be definitively linked to the intervention (Preston et al., 2018). Additionally, a small study was conducted to evaluate the safety of *L. casei* shirota when treating diarrhea occurring in critically ill children, in which no safety signals were observed (Srinivasan et al., 2006).

#### HIV/AIDS-Associated Diarrhea

Gastrointestinal diseases are a common disorder in patients suffering from human immunodeficiency virus (HIV) and/or acquired immunodeficiency disorder (AIDS), with roughly 40% of HIV/AIDS patients suffering from GI related hyponatremia in certain areas of the world (Shu et al., 2018). Diarrhea in HIV/AIDS affected individuals can be caused by a variety of opportunistic infections or noninfectious causes linked to treatment regimens (Dikman et al., 2015). Current treatment for patients suffering from AIDS-AD involve antisecretory agents and/or fecal microbiota transplantation therapy (Dikman et al., 2015; Ouyang, 2020). Several promising studies have provided insight into utilizing probiotics as an affordable and accessible option to combat diarrhea in patients with HIV/AIDS induced diarrhea. Probiotic yogurts have been historically utilized in Africa to ease HIV/AIDS AD (Reid, 2010; Whaling, 2012). In a 2008 study conducted in Nigeria, yogurt with probiotics *Lactobacillus delbruekii* subsp. *bulgaricus* and *S. thermophilus*, or *L. rhamnosus GR-1* and *L. reuteri RC-14* resolved diarrheal symptoms in 12/12 patients after 15 days of consumption compared to 2/12 in the control group (Anukam et al., 2008). However, Salminen et al. (2004) study demonstrated little to no difference between experimental and control groups (Salminen et al., 2004). A double-blinded, randomized, placebo-controlled trial involving 44 patients over 12 weeks utilizing molecular sequencing techniques to analyze changes in the gut microbiome following *S. boulardii* administration demonstrated a significant reduction in pathogenic bacterial species of the Clostridiaceae family and a reduction in inflammatory biomarkers (Villar-García, 2017).

### Drug Interactions

The co-administration of probiotics with orally administered drugs warrants further investigation. Although oral administration of drugs is the most convenient, economical, and common route of administration, subsequent interactions with food, co-administered drugs, or microbiota may influence absorption and bioavailability. Gut microbiota are known to produce a diverse array of enzymes capable of metabolizing nutrients and drugs (Claus et al., 2011), which could alter the structure of the parent compound and subsequent membrane diffusion, active transport into the bloodstream, and/or efficacy. The therapeutic activity of lactulose depends on the metabolism by intestinal bacteria such as *Lactobacillus* (Sahota et al., 1982), which are also employed as probiotics. Interestingly, *Lactobacillus* metabolites have also been shown to compete for hepatic uptake of drugs such as simvastain, thereby altering the pharmacodynamics (Kaddurah-Daouk et al., 2011). While significant data outlining the relationship between gut microbiota and drug pharmacokinetics and pharmacodynamics (PK/PD) has been reported (Yoo et al., 2014; Swansan, 2015; Zhang et al., 2018), insufficient studies have been conducted to determine the drug interactions associated with the co-administration of probiotics. One animal study determined the administration of *E. coli* Nissle 1917 (ATCC 25922) altered the PK of amidarone absorption in rats and led to a 43% increase in exposure (Matuskova et al., 2014).

## Conclusion

Natural microbial colonization occurs after birth and may vary significantly based upon environmental factors and antibiotic administration (Conlon and Bird, 2014). The gut microbiota has gained interest in recent years with respect to probiotic dietary interventions and the regulation of intestinal homeostasis. The metabolic importance of the gut microbiota to nutrient and drug pharmacokinetics underscores the potential of probiotic use for preventive or therapeutic applications in various gastrointestinal disorders. From a mechanistic perspective, probiotics have been shown to strengthen the gut epithelial barrier and reduce inflammation.

Although promising results have been demonstrated for a variety of probiotics undergoing clinical trials to treat complex gastrointestinal and inflammatory diseases, the traditional drug development paradigm associated with preclinical and clinical studies is lacking. Since probiotics are not typically under premarket evaluation by the FDA, the formulation, dosing regimen, mechanism(s) of action, and clinical pharmacology are not readily available in a package insert for healthcare providers. Despite the variability, safety considerations will generally favor a commercially available probiotic approach over the administration of fecal transplantation. An increasing number of clinical trials have indicated improved patient outcomes relative to probiotic use to treat IBS, NEC, antibiotic and HIV AD, and AG. To date, well-controlled clinical studies to clearly document the prophylactic and therapeutic effects of probiotics are limited, which illustrates the numerous gaps relative to the systematic evaluation of species, formulations, and dosing relative to disease indication.

Despite the drawbacks, clinicians recognize the importance of gut microbiota in disruption of several diseases and have been exploring the use of probiotics to restore a ‘healthy’ microbiome. Often patients either self-administer or a healthcare provider indicates probiotic use to restore gut microbiota, but the clinical outcomes are challenging to extrapolate given the heterogeneity of probiotics relative to species, strain(s), purity, formulation, and manufacturer. The concomitant use of probiotics, antibiotics, and other drug classes further alters the pharmacokinetic and pharmacodynamic profile of treatment regimens, while introducing the potential for drug-drug interactions and should be considered relative to patient polypharmacy (Zhang et al., 2018). The myriad of descriptive and observational studies reviewed underscore the need for randomized controlled trials with clearly defined formulations, species, strain(s), dosing regimens, pharmacodynamic endpoints, clinical outcomes and biostatistical analyses.

## Author Contributions

DR is a student of Newburgh Free Academy, Newburgh, New York who participated in a student research program at the United States Military Academy. All authors contributed to manuscript drafts and revisions, read, and approved the submitted version.

## Author Disclaimer

The views expressed herein are those of the authors and do not reflect the position of the United States Military Academy, the Department of the Army, or the Department of Defense.

## Conflict of Interest

The authors declare that the research was conducted in the absence of any commercial or financial relationships that could be construed as a potential conflict of interest.

## Publisher’s Note

All claims expressed in this article are solely those of the authors and do not necessarily represent those of their affiliated organizations, or those of the publisher, the editors and the reviewers. Any product that may be evaluated in this article, or claim that may be made by its manufacturer, is not guaranteed or endorsed by the publisher.

## References

[B1] Ables A. Simon I. Melton E. (2007). Update on *Helicobacter pylori* treatment. *Am. Fam. Physician.* 75 351–358. 17304866

[B2] Aidy S. E. Hooiveld G. Tremaroli V. Backhed F. Kleerebezem M. (2013). The gut microbiota and mucosal homeostasis: colonized at birth or at adulthood, does it matter? *Gut Microbes* 4 118–124. 10.4161/gmic.23362 23333858 PMC3595071

[B3] Alby K. Nachamkin I. (2016). Gastrointestinal infections. *Microbiol. Spectr.* 4 10.1128/microbiolspec.DMIH2-0005-2015 27337464

[B4] AlFaleh K. Anabrees J. (2014). Probiotics for prevention of necrotizing enterocolitis in preterm infants. *Evid. Based Child Health* 9 584–671. 10.1002/ebch.1976 25236307

[B5] Altwegg M. Schnack J. Zbinden R. (1995). Influence of Saccharomyces boulardii on Aeromonas hemolysin. *Med. Microbiol. Lett.* 4 417–425.

[B6] Anderson R. C. (2010). Lactobacillus plantarum MB452 enhances the function of the intestinal barrier by increasing the expression levels of genes involved in tight junction formation. *BMC Microbiol.* 10:316. 10.1186/1471-2180-10-316 21143932 PMC3004893

[B7] Anukam K. Osazuwa E. Osadolor H. Bruce A. Reid G. (2008). Yogurt containing probiotic Lactobacillus rhamnosus GR-1 and L. reuteri RC-14 helps resolve moderate diarrhea and increases CD4 count in HIV/AIDS patients. *J. Clin. Gastroenterol.* 42 239–243. 18223503 10.1097/MCG.0b013e31802c7465

[B8] Auclair J. Frappier M. Millette M. (2015). Lactobacillus acidophilus CL1285, Lactobacillus casei LBC80R, and Lactobacillus rhamnosus CLR2 (Bio-K+): characterization, manufacture, mechanisms of action, and quality control of a specific probiotic combination for primary prevention of clostridium dif. *Clin. Infect. Dis.* 60(suppl_2) S135–S143. 10.1093/cid/civ179 25922399

[B9] Azaïs-Braesco V. Bresson J. Guarner F. Corthier G. (2010). Not all lactic acid bacteria are probiotics, . but some are. *Br. J. Nutr.* 103 1079–1081. 10.1017/S0007114510000723 20230653

[B10] Aziz I. Simrén M. (2021). The overlap between irritable bowel syndrome and organic gastrointestinal diseases. *Lancet Gastroenterol. Hepatol.* 6 139–148. 10.1016/S2468-1253(20)30212-0 33189181

[B11] Bäckhed F. Ley R. Sonnenburg J. Peterson D. Gordon J. (2005). Host-bacterial mutualism in the human intestine. *Science* 307 1915–1920. 10.1126/science.1104816 15790844

[B12] Barbut F. Meynard J. (2002). Managing antibiotic associated diarrhoea. *Br. Med. J.* 324 1345–1346. 10.1136/bmj.324.7350.1345 12052785 PMC1123310

[B13] Bäumler A. J. Sperandio V. (2016). Interactions between the microbiota and pathogenic bacteria in the gut. *Nature* 535 85–93. 10.1038/nature18849 27383983 PMC5114849

[B14] Belda I. Ruiz J. Santos A. Van Wyk N. Pretorius I. S. (2019). Saccharomyces cerevisiae. *Trends Gemet.* 35 956–957. 10.1015/j.tig.2019.08.009 31630852

[B15] Bermudez-Brito M. Plaza-Díaz J. Muñoz-Quezada S. Gómez-Llorente C. Gil A. (2012). Probiotic mechanisms of action. *Ann. Nutr. Metab.* 61 160–174. 10.1159/000342079 23037511

[B16] Blum J. E. (2013). Frequent replenishment sustains the beneficial microbiome of Drosophila melanogaster. *mBio* 4:e860–13. 10.1128/mBio.00860-13 24194543 PMC3892787

[B17] Boirivant M. Strober W. (2007). The mechanism of action of probiotics. *Curr. Opin. Gastroenterol.* 23 679–692. 10.1097/MOG.0b013e3282f0cffc 17906447

[B18] Castagliuolo I. Lamont J. T. Nikulasson S. T. Pothoulakis C. (1996). Saccharomyces boulardii protease inhibits Clostridium difficile toxin A effects in the rat ileum. *Infect. Immun.* 64 5225–5232. 10.1128/IAI.64.12.5225-5232.1996 8945570 PMC174512

[B19] Chandler J. A. (2012). Yeast communities of diverse *Drosophila* species: comparison of two symbiont groups in the same hosts. *Appl. Environ. Microbiol.* 78 7327–7336. 22885750 10.1128/AEM.01741-12 PMC3457106

[B20] Chang E. B. Martinez-Guryn K. (2019). Small intestinal microbiota: the neglected stepchild needed for fat digestion and absorption. *Gut Microb.* 10 235–240. 10.1080/19490976.2018.1502539 30136893 PMC6546320

[B21] Chang H. Y. (2017). Multiple strains probiotics appear to be the most effective probiotics in the prevention of necrotizing enterocolitis and mortality: An updated meta-analysis. *PLoS One* 12:e0171579. 10.1371/journal.pone.0171579 28182644 PMC5300201

[B22] Chey W. D. Leontidis G. I. Howden C. W. Moss S. F. (2017). ACG clinical guideline: treatment of *Helicobacter* pylori infection. *Am. J. Gastroenterol.* 112 212–238. 10.1038/ajg.2016.563 28071659

[B23] Cindoruk M. Erkan G. Karakan T. Dursun A. Unal S. (2007). Efficacy and Safety of Saccharomyces boulardii in the 14-day Triple Anti-*Helicobacter* pylori therapy: a prospective randomized placebo-controlled double-blind study. *Helicobacter* 12 309–316. 10.1111/j.1523-5378.2007.00516.x 17669103

[B24] Ciorba M. A. Riehl T. E. Rao M. S. Moon C. Ee X. Nava G. M. (2012). Lactobacillus probiotic protects intestinal epithelium from radiation injury in a TLR-2/cyclo-oxygenase-2-dependent manner. *Gut* 61 829–838. 10.1136/gutjnl-2011-300367 22027478 PMC3345937

[B25] Claus S. P. Ellero S. L. Berger B. Krause L. Bruttin A. Molina J. (2011). Colonization-induced host-gut microbial metabolic interaction. *mBio* 2:e271–10. 10.1128/mBio.00271-10 21363910 PMC3045766

[B26] Collado M. C. (2006). Protection mechanism of probiotic combination against human pathogens: *in vitro* adhesion to human intestinal mucus. *Asia Pac. J. Clin. Nutr.* 15 570–575. 17077078

[B27] Conlon M. Bird A. (2014). The impact of diet and lifestyle on gut microbiota and human health. *Nutrients* 7 17–44. 10.3390/nu7010017 25545101 PMC4303825

[B28] Cornick S. Tawiah A. Chadee K. (2015). Roles and regulation of the mucus barrier in the gut. *Tissue Barriers* 3:e982426. 10.4161/21688370.2014.982426 PMC4372027 25838985

[B29] Costeloe K. Hardy P. Juszczak E. Wilks M. Millar M. R. (2016). Bifidobacterium breve BBG-001 in very preterm infants: a randomised controlled phase 3 trial. *Lancet* 387 649–660. 10.1016/S0140-6736(15)01027-2 26628328

[B30] Coyle C. M. Varughese J. Weiss L. M. Tanowitz H. B. (2012). Blastocystis: to treat or not to treat. *Clin. Infect. Dis.* 54 105–110. 10.1093/cid/cir810 22075794

[B31] Cutting S. M. (2011). Bacillus probiotics. *Food Microbiol.* 28 214–220. 10.1016/j.fm.2010.03.007 21315976

[B32] Defrees D. N. (2017). Irritable bowel syndrome: epidemiology, pathophysiology, diagnosis, and treatment. *Primary Care* 44 655–671. 10.1016/j.pop.2017.07.009 29132527

[B33] Deng K. Chen T. Wu Q. Xin H. Wei Q. Hu P. (2015). *In vitro* and *in vivo* examination of anticolonization of pathogens by Lactobacillus paracasei FJ861111.1. *J. Dairy Sci.* 98 6759–6766. 10.3168/jds.2015-9761 26254535

[B34] Deng L. W. (2021). New insights into the interactions between Blastocystis, the gut microbiota, and host immunity. *PLoS Pathog.* 17:e100925. 10.1371/journal.ppat.1009253 33630979 PMC7906322

[B35] Dikman A. Schonfeld E. Srisarajivakul N. Poles M. (2015). Human immunodeficiency virus-associated diarrhea: still an issue in the era of antiretroviral therapy. *Dig. Dis. Sci.* 60 2236–2245. 25772777 10.1007/s10620-015-3615-y PMC4499110

[B36] Dini C. Bolla P. de Urraza P. (2016). Treatment of in vitro enterohemorrhagic *Escherichia coli* infection using phage and probiotics. *J. Appl. Microbiol.* 121 78–88. 10.1111/jam.13124 26969848

[B37] Dinleyici E. C. Eren M. Dogan N. Reyhanioglu S. Yargic Z. A. Vandenplas Y. (2010). Clinical efficacy of Saccharomyces boulardii or metronidazole in symptomatic children with Blastocystis hominis infection. *Nat. Library Med.* 108 541–545. 10.1007/s00436-010-2095-4 20922415

[B38] Du Y. Su T. Fan J. Lu Y. Zheng P. Li X. (2012). Adjuvant probiotics improve the eradication effect of triple therapy for *Helicobacter* pylori infection. *World J. Gastroenterol.* 18 6302–6307. 23180952 10.3748/wjg.v18.i43.6302 PMC3501780

[B39] Ducluzeau R. Bensaada M. (1982). [Comparative effect of a single or continuous administration of “Saccharomyces boulardii” on the establishment of various strains of “candida” in the digestive tract of gnotobiotic mice]. *Ann. Microbiol. (Paris)* 133 491–501. 6762128

[B40] Eetemadi A. Rai N. Pereira B. M. Kim M. Schmitz H. Tagkopoulos I. (2020). The computational diet: a review of computational methods across diet. Microbiome, and Health. *Front. Microbiol.* 11:393. 10.3389/fmicb.2020.00393 32318028 PMC7146706

[B41] Elvers K. T. (2020). Antibiotic-induced changes in the human gut microbiota for the most commonly prescribed antibiotics in primary care in the UK: a systematic review. *BMJ Open* 10:e035677. 10.1136/bmjopen-2019-035677 32958481 PMC7507860

[B42] Eugenia Bezirtzoglou E. S. (2011). Immunology and probiotic impact of the newborn and young children intestinal microflora. *Anaerobe* 17 369–374. 10.1016/j.anaerobe.2011.03.010 21515397

[B43] Farré-Maduell E. Casals-Pascual C. (2019). The origins of gut microbiome research in Europe: from Escherich to Nissle. *Hum. Microb. J.* 14:100065. 10.1016/j.humic.2019.100065

[B44] Fijan S. Sulc D. Steyer A. (2018). Study of the *In Vitro* antagonistic activity of various single-strain and multi-strain probiotics against *Escherichia coli*. *Int. J. Environ. Res. Public Health* 15:1539. 10.3390/ijerph15071539 30036977 PMC6069398

[B45] Fjalstad J. Esaiassen E. Juvet L. K. van den Anker J. N. Klingenberg C. (2018). Antibiotic therapy in neonates and impact on gut microbiota and antibiotic resistance development: a systematic review. *J. Antimicrob. Chemother.* 73 569–580. 10.1093/jac/dkx426 29182785

[B46] Fredua-Agyeman M. Stapleton P. Basit A. Beezer A. Gaisford S. (2017). In vitro inhibition of Clostridium difficile by commercial probiotics: a microcalorimetric study. *Int. J. Pharm.* 517 96–103. 10.1016/j.ijpharm.2016.12.005 27923699

[B47] Freedman S. B. Xie J. Nettel-Aguirre A. Pang X.-L. Chui L. Williamson-Urquhart S. (2020). A randomized trial evaluating virus-specific effects of a combination probiotic in children with acute gastroenteritis. *Nat. Commun.* 11:2533. 10.1038/s41467-020-16308-3 32439860 PMC7242434

[B48] Gasbarrini G. Bonvicini F. Gramenzi A. (2016). Probiotics history. *J. Clin. Gastroenterol.* 50(Suppl. 2) S116–S119. 10.1097/MCG.0000000000000697 27741152

[B49] Gehart H. Clevers H. (2019). Tales from the crypt: new insights into intestinal stem cells. *Nat. Rev. Gastroenterol. Hepatol.* 16 19–34. 10.1038/s41575-018-0081-y 30429586

[B50] Gensollen T. Iyer S. S. Kasper D. L. Blumberg R. S. (2016). How colonization by microbiota in early life shapes the immune system. *Science* 352 539–544. 10.1126/science.aad9378 27126036 PMC5050524

[B51] Gibson M. K. Crofts T. S. Dantas G. (2015). Antibiotics and the developing infant gut microbiota and resistome. *Curr. Opin. Microbiol.* 27 51–56. 10.1016/j.mib.2015.07.007 26241507 PMC4659777

[B52] Grandview Research (2019). *Probiotics Market Size, Share & Trends Analysis Report By Product (Food & Beverages, Dietary Supplements), By Ingredient (Bacteria, Yeast), By End Use, By Distribution Channel, And Segment Forecasts, 2019 - 2025.* Available online at: https://www.grandviewresearch.com/industry-analysis/probiotics-market (accessed June 05, 2021)

[B53] Graves N. S. (2013). Acute gastroenteritis. *Natl. Library Med.* 40 727–741. 10.1016/j.pop.2013.05.006 23958366 PMC7119329

[B54] Harmon E. J. (2017). *Bacteriological Analytical Manual (BAM) Chapter 16: Clostridium Perfringens.* Silver Spring, MD: Food and Drug Administration.

[B55] Harris L. Baffy N. (2017). Modulation of the gut microbiota: a focus on treatments for irritable bowel syndrome. *Postgrad Med.* 129 872–888. 28936910 10.1080/00325481.2017.1383819

[B56] Hartman S. Brown E. Loomis E. Russell H. A. (2019). Gastroenteritis in children. *Am. Family Phys.* 99 159–165. 30702253

[B57] He Q. W. (2013). Microbial fingerprinting detects intestinal microbiota dysbiosis in Zebrafish models with chemically-induced enterocolitis. *BMC Microbiol.* 13:289. 10.1186/1471-2180-13-289 24325678 PMC4029296

[B58] Hickson M. (2011). Probiotics in the prevention of antibiotic-associated diarrhoea and Clostridium difficile infection. *Therapeutic Adv. Gastroenterol.* 4 185–197. 10.1177/1756283X11399115 21694803 PMC3105609

[B59] Higuchi K. Maekawa T. Nakagawa K. Chouno S. Hayakumo T. Tomono N. (2006). Efficacy and safety of *Helicobacter* pylori eradication therapy with omeprazole, amoxicillin and high- and low-dose clarithromycin in Japanese patients: a randomised, double-blind, multicentre study. *Clin. Drug Investig.* 26 403–414. 10.2165/00044011-200626070-00002 17163273

[B60] Holt J. G. Krieg N. A. Sneath P. H. Staley J. T. Williams S. T. (2000). *Bergey’s Manual of Determinative Bacteriology*, 9th Edn. Philadelphia, PA: Williams & Wilkins.

[B61] Hooi J. L. (2017). Global Prevalence of *Helicobacter* pylori infection: systematic review and meta-analysis. *Gastroenterology* 153 420–429. 10.1053/j.gastro.2017.04.022 28456631

[B62] Hu L. Zhou M. Young A. Zhao W. Yan Z. (2019). In vivo effectiveness and safety of probiotics on prophylaxis and treatment of oral candidiasis: a systematic review and meta-analysis. *BMC Oral Health* 19:140. 10.1186/s12903-019-0841-2 31291932 PMC6621984

[B63] Humphries R. M. (2015). Practical guidance for clinical microbiology laboratories: diagnosis of bacterial gastroenteritis. *Clin. Microbiol. Rev.* 28 3–31. 10.1128/CMR.00073-14 25567220 PMC4284301

[B64] Hyun H. H. (1983). Ultrastructure and extreme heat resistance of spores from thermophilic Clostridium species. *J. Bacteriol.* 156 1332–1337. 10.1128/JB.156.3.1332-1337.1983 6643392 PMC217984

[B65] International Probiotics Association (2017). *Council for Responsible Nutrition.* Available online at: https://www.crnusa.org/sites/default/files/pdfs/CRN-IPA-Best-Practices-Guidelines-for-Probiotics.pdf (accessed May 10, 2021)

[B66] Jiang H. P. (2009). Cytokine/Jak/Stat signaling mediates regeneration and homeostasis in the *Drosophila* midgut. *Cell* 137 1343–1355. 10.1016/j.cell.2009.05.014 19563763 PMC2753793

[B67] Joint Food and Agriculture Organization/World Health Organization Working Group (2002). *Joint FAO/WHO Working Group Report on Drafting Guidelines for the Evaluation of Probiotics in Food.* Geneva: World Health Organization.

[B68] Jungersen M. Wind A. Johansen E. Christensen J. E. Stuer-Lauridsen B. Eskesen D. (2014). The science behind the probiotic strain Bifidobacterium animalis subsp. lactis BB-12. *Microorganisms* 2 92–110. 10.3390/microorganisms2020092 27682233 PMC5029483

[B69] Kaddurah-Daouk R. Baillie R. Zhu H. Zeng Z. Wiest M. Nguyen U. (2011). Enteric microbiome metabolites correlate with response to simvastatin treatment. *PLoS One* 6:e25482. 10.1371/journal.pone.0025482 22022402 PMC3192752

[B70] Kamareddine L. Najjar H. Umar Sohail M. Abdulkader H. Al-Asmakh M. (2020). The microbiota and gut-related disorders: insights from animal models. *Cells* 9:2401. 10.3390/cells9112401 33147801 PMC7693214

[B71] Karska-Wysocki B. Bazo M. Smoragiewicz W. (2010). Antibacterial activity of Lactobacillus acidophilus and Lactobacillus casei against methicillin-resistant Staphylococcus aureus (MRSA). *Microbiol. Res.* 165 674–686. 10.1016/j.micres.2009.11.008 20116228

[B72] Kim M. J. Ku S. Kim S. Y. Lee H. H. Jin H. Kang S. (2018). Safety Evaluations of Bifidobacterium bifidum BGN4 and Bifidobacterium longum BORI. *Int. J. Mol. Sci.* 19:1422. 10.3390/ijms19051422 29747442 PMC5983828

[B73] Kluijfhout S. Trieu T. Vandenplas Y. (2020). Efficacy of the probiotic probiotical confirmed in acute gastroenteritis. *Pediatr. Gastroenterol. Hepatol. Nutr.* 23 464–471. 10.5223/pghn.2020.23.5.464 32953642 PMC7481057

[B74] Kotowska M. Albrecht P. Szajewska H. (2005). Saccharomyces boulardii in the prevention of antibiotic-associated diarrhoea in children: a randomized double-blind placebo-controlled trial. *Alimentary Pharmacol. Therapeutics* 21 583–590. 10.1111/j.1365-2036.2005.02356.x 15740542

[B75] Koutsokali M. Valahas M. (2020). Anaerobic and aerobic respiration in yeast: small-scale variations on a classic laboratory activity. *J. Chem. Educ.* 97 1041–1047. 10.1021/acs.jchemed.9b00994

[B76] Kovács ÁT. (2019). Bacillus subtilis. *Trends Microbiol.* 27 724–725. 10.1016/j.tim.2019.03.008 31000489

[B77] Laukens D. Brinkman B. M. Raes J. De Vos M. Vandenabeele P. (2016). Heterogeneity of the gut microbiome in mice: guidelines for optimizing experimental design. *FEMS Microbiol. Rev.* 40 117–132. 10.1093/femsre/fuv036 26323480 PMC4703068

[B78] Lepczyńska M. Dzika E. (2019). The influence of probiotic bacteria and human gut microorganisms causing opportunistic infections on Blastocystis ST3. *Gut Pathogens* 11 2–11. 10.1186/s13099-019-0287-8 30815037 PMC6376780

[B79] Ley R. E. (2006). Ecological and evolutionary forces shaping microbial diversity in the human intestine. *Cell* 124 837–848. 10.1016/j.cell.2006.02.017 16497592

[B80] Lu C. Sang J. He H. Wan X. W. Lin Y. Li L. (2016). Probiotic supplementation does not improve eradication rate of *Helicobacter pylori* infection compared to placebo based on standard therapy: a meta-analysis. *Nat. Sci. Rep.* 6:23522. 10.1038/srep23522 26997149 PMC4800733

[B81] Lu Y. Z. (2019). Screening of intestinal peristalsis-promoting probiotics based on a zebrafish model. *Food Funct.* 10 2075–2082. 10.1039/c8fo02523a 30911742

[B82] Mack D. R. Michail S. Wei S. McDougall L. Hollingsworth M. A. (1999). Probiotics inhibit enteropathogenic E. coli adherence *in vitro* by inducing intestinal mucin gene expression. *Am. J. Physiol.* 276 G941–G950. 10.1152/ajpgi.1999.276.4.G941 10198338

[B83] MacPherson C. W. (2018). Gut bacterial microbiota and its resistome rapidly recover to basal state levels after short-term amoxicillin-clavulanic acid treatment in healthy adults. *Sci. Rep.* 8:11192. 10.1038/s41598-018-29229-5 30046129 PMC6060159

[B84] Mada P. K. Alam M. U. (2020). *Clostridium Difficile.* Available online at: https://www.ncbi.nlm.nih.gov/books/NBK431054 (accessed December 15, 2020)

[B85] Madigan M. T. (2018). *Brock Biology of Microorganisms.* London: Pearson.

[B86] Mann E. R. Landy J. D. Bernardo D. Peake S. T. Hart A. L. Al-Hassi H. O. (2013). Intestinal dendritic cells: their role in intestinal inflammation, manipulation by the gut microbiota and differences between mice and men. *Immunol. Lett.* 150 30–40. 10.1016/j.imlet.2013.01.007 23352670

[B87] Matuskova Z. Anzenbacherova E. Vecera R. Tlaskalova-Hogenova H. Kolar M. Anzenbacher P. (2014). Administration of a probiotic can change drug pharmacokinetics: effect of E. coli Nissle 1917 on amidarone absorption in rats. *PLoS One* 9:e87150. 10.1371/journal.pone.0087150 24505278 PMC3914806

[B88] McCullough M. J. Clemons K. V. McCusker J. H. Stevens D. A. (1998). Species identification and virulence attributes of Saccharomyces boulardii (nom. inval.). *J. Clin. Microbiol.* 36 2613–2617. 10.1128/JCM.36.9.2613-2617.1998 9705402 PMC105172

[B89] McFarland L. (1996). Saccharomyces boulardiiIs Not Saccharomyces cerevisiae. *Clin. Infect. Dis.* 22 200–201. 10.1093/clinids/22.1.200 8825013

[B90] McFarland L. (1998). Epidemiology, risk factors and treatments for antibiotic-associated diarrhea. *Dig. Dis.* 16 292–307. 10.1159/000016879 9892789

[B91] McFarland L. V. (2006). Meta-analysis of probiotics for the prevention of antibiotic associated diarrhea and the treatment of Clostridium difficile disease. *Am. J. Gastroenterol.* 101 812–822. 10.1111/j.1572-0241.2006.00465.x 16635227

[B92] McFarland L. V. (2007). Diarrhoea associated with antibiotic use. *BMJ (Clin. Res. ed.)* 335 54–55. 10.1136/bmj.39255.829120.47 17626915 PMC1914493

[B93] Metchnikoff É (1907). *The Prolongation of Life: Optimistic Studies. (P. Chalmers Mitchell, Trans.).* New York, NY: Springer.

[B94] Metras B. N. (2020). Assessment of commercial companion animal kefir products for label accuracy of microbial composition and quantity. *J. Anim. Sci.* 98:skaa301. 10.1093/jas/skaa301 32914845 PMC7523595

[B95] Metras B. N. Holle M. J. Parker V. J. Miller M. J. Swanson K. S. (2021). Commercial kefir products assessed for label accuracy of microbial composition and density. *JDS Comms.* 2 87–91. 10.3168/jdsc.2020-0056 PMC9623786 36339502

[B96] Mills J. P. (2018). Probiotics for prevention of Clostridium difficile infection. *Curr. Opin. Gastroenterol.* 34 3–10. 10.1097/MOG.0000000000000410 29189354 PMC6335148

[B97] Mohsin M. Guenther S. Schierack P. Tedin K. Wieler L. H. (2015). Probiotic *Escherichia coli* Nissle 1917 reduces growth, Shiga toxin expression, release and thus cytotoxicity of enterohemorrhagic *Escherichia coli*. *Int. J. Med. Microbiol.* 305 20–26. 10.1016/j.ijmm.2014.10.003 25465158

[B98] National Institutes of Health, National Center for Complementary and Integrative Health (2021). *Probiotics: What You Need To Know.* Bethesda, MD: National Institutes of Health.

[B99] National Institutes of Health (2019). *Probiotics Fact Sheet for Consumer.* Bethesda, MD: National Institutes of Health.

[B100] National Institutes of Health, Office of Dietary Supplements (1994). *Dietary Supplement Health and Education Act of 1994 Public Law 103-417 103rd Congress.* Available online at: https://ods.od.nih.gov/about/dshea_wording.aspx#sec3 (accessed May 7, 2021).

[B101] Natividad J. M. Verdu E. F. (2013). Modulation of intestinal barrier by intestinal microbiota: pathological and therapeutic implications. *Pharmacol. Res.* 69 42–51. 10.1016/j.phrs.2012.10.007 23089410

[B102] Neu J. Walker W. A. (2011). Necrotizing enterocolitis. *N. Engl. J. Med.* 364 255–264. 10.1056/NEJMra1005408 21247316 PMC3628622

[B103] Ng K. M.-D. (2019). Recovery of the gut microbiota after antibiotics depends on host diet, community context, and environmental reservoirs. *Cell Host Microbe* 26 650–665.e4. 10.1016/j.chom.2019.10.011 31726029 PMC8276089

[B104] Noah T. K. Donahue B. Shroyer N. F. (2011). Intestinal development and differentiation. *Exp. Cell Res.* 317 2702–2710. 10.1016/j.yexcr.2011.09.006 21978911 PMC3210330

[B105] Oehlers S. H. (2011). A chemical enterocolitis model in zebrafish larvae that is dependent on microbiota and responsive to pharmacological agents. *Dev. Dyn.* 240 288–298. 10.1002/dvdy.22519 21181946

[B106] Ohland C. L. Macnaughton W. K. (2010). Probiotic bacteria and intestinal epithelial barrier function. *Am. J. Physiol. Gastrointest Liver Physiol.* 298 G807–G819. 20299599 10.1152/ajpgi.00243.2009

[B107] Ouyang J. I. (2020). Treating From the Inside Out: Relevance of Fecal Microbiota Transplantation to Counteract Gut Damage in GVHD and HIV Infection. *Front. Med. (Lausanne)* 7:421. 10.3389/fmed.2020.00421 32850913 PMC7423874

[B108] Pavan S. Desreumaux P. Mercenier A. (2003). Use of mouse models to evaluate the persistence, safety, and immune modulation capacities of lactic acid bacteria. *Clin. Diagn Lab Immunol.* 10 696–701. 10.1128/cdli.10.4.696-701.2003 12853407 PMC164262

[B109] Pothoulakis C. (2009). Review article: anti-inflammatory mechanisms of action of Saccharomyces boulardii. *Alimentary Pharmacol. Therapeutics* 30 826–833. 10.1111/j.1365-2036.2009.04102.x 19706150 PMC2761627

[B110] Preston K. Krumian R. Hattner J. Montigny D. Stewart M. Gaddam S. (2018). Lactobacillus acidophilus CL1285, Lactobacillus casei LBC80R and Lactobacillus rhamnosus CLR2 improve quality-of-life and IBS symptoms: a double-blind, randomised, placebo-controlled study. *Benef. Microbes* 9 697–706. 29888656 10.3920/BM2017.0105

[B111] Probiotics Market Size Share and Trends Analysis Report By Product (2019). *Grand View Research.* Available online at: https://www.grandviewresearch.com/press-release/global-probiotics-market (accessed January 21, 2021)

[B112] Rawls J. F. (2006). Reciprocal gut microbiota transplants from zebrafish and mice to germ-free recipients reveal host habitat selection. *Cell* 127 423–433. 10.1016/j.cell.2006.08.043 17055441 PMC4839475

[B113] Reid G. (2010). The potential role for probiotic yogurt for people living with HIV/AIDS. *Gut Microbes* 1 411–414. 10.4161/gmic.1.6.14079 21468226 PMC3056109

[B114] Reid G. Gadir A. Dhir R. (2019). Probiotics: reiterating what they are and what they are not. *Front. Microbiol.* 10:424. 10.3389/fmicb.2019.00424 30930863 PMC6425910

[B115] Rieko M. Osamu H. Yosuke S. Atsushi M. Yuji N. (2020). Effectiveness of including probiotics to *Helicobacter* pylori eradication therapies. *J. Clin. Biochem. Nutr.* 67 102–104. 32801475 10.3164/jcbn.20-37 PMC7417795

[B116] Ringel-Kulka T. Palsson O. Maier D. Carroll I. Galanko J. Leyer G. (2015). *Clinical trial: Probiotic Bacteria Lactobacillus acidophilus NCFM and Bifidobacterium lactis Bi-07 Versus Placebo for the Symptoms of Bloating in Patients with Functional Bowel Disorders.* Bethesda, MD: National Center for Biotechnology Information. 10.1097/MCG.0b013e31820ca4d6 PMC4372813 21436726

[B117] Roberts T. Ellis J. Harkness J. Marriott D. Stark D. (2014). Treatment failure in patients with chronic Blastocystis infection. *J. Med. Microbiol.* 63 252–257. 10.1099/jmm.0.065508-0 24243286

[B118] Saavedra J. M. Bauman N. A. Perman J. A. Yolken R. H. Oung I. (1994). Feeding of Bifidobacterium bifidum and Streptococcus thermophilus to infants in hospital for prevention of diarrhoea and shedding of rotavirus. *Lancet* 344 1046–1049. 10.1016/s0140-6736(94)91708-6 7934445

[B119] Sahota S. S. Bramley P. M. Menzies I. S. (1982). The fermentation of lactulose by colonic bacteria. *J. Gen. Microbiol.* 128 319–325. 10.1099/00221287-128-2-319 6804597

[B120] Salminen M. Tynkkynen S. Rautelin H. Poussa T. Saxelin M. Ristola M. (2004). The efficacy and safety of probiotic Lactobacillus rhamnosus GG on prolonged, noninfectious diarrhea in HIV Patients on antiretroviral therapy: a randomized, placebo-controlled, crossover study. *HIV Clin. Trials* 5 183–191. 15472792 10.1310/6F83-N39Q-9PPP-LMVV

[B121] Sandra M. Tallent A. K. (2020). *Bacteriological Analytical Manual (BAM) Chapter 14: Bacillus Cereus.* Silver Spring, MA: Food and Drug Administration.

[B122] Saxelin M. (2008). Probiotic formulations and applications, the current probiotics market, and changes in the marketplace: a European perspective. *Clin. Infect. Dis.* 46 S144–S151. 10.1086/523337 18181728

[B123] Schaik W. V. (2015). The human gut resistome. *Philos Trans. R. Soc. Lond B Biol. Sci.* 370 e1–e9. 10.1098/rstb.2014.0087 25918444 PMC4424436

[B124] Sekar U. Shanthi M. (2013). Blastocystis: consensus of treatment and controversies. *Trop. Parasitol.* 3 35–39. 10.4103/2229-5070.113901 23961439 PMC3745668

[B125] Sender R. Fuchs S. Milo R. (2016). Are we really vastly outnumbered? Revisiting the ratio of bacterial to host cells in humans. *Cell* 164 337–340. 10.1016/j.cell.2016.01.013 26824647

[B126] Shelby R. Janzow G. Mashburn-Warren L. Galley J. Tengberg N. Navarro J. (2020). A novel probiotic therapeutic in a murine model of Clostridioides difficile colitis. *Gut Microbes* 12:1814119. 10.1080/19490976.2020 PMC7524353 32954922

[B127] Shu Z. Tian Z. Chen J. Ma J. Abudureyimu A. Qian Q. (2018). HIV/AIDS-related hyponatremia: an old but still serious problem. *Ren. Fail.* 40 68–74. 29299949 10.1080/0886022X.2017.1419975 PMC6014325

[B128] Sichetti M. Marco S. D. Pagiotti R. Traina G. Pietrella D. (2018). Anti-inflammatory effect of multistrain probiotic formulation (L. rhamnosus. B. lactis, and B. longum). *Nutrition* 53 95–102. 10.1016/j.nut.2018.02.005 29674267

[B129] Silverman M. A. Konnikova L. Gerber J. S. (2017). Impact of antibiotics on necrotizing enterocolitis and antibiotic-associated diarrhea. *Gastroenterol. Clin. North Am.* 46 61–76. 28164853 10.1016/j.gtc.2016.09.010 PMC5314436

[B130] Sinclair J. B. (2016). Blastocystis hominis: the fascinating enigma. *Aust. J. Med. Sci.* 37 3–4.

[B131] Sniffen J. C. McFarland L. V. Evans C. T. Goldstein E. J. (2018). Choosing an appropriate probiotic product for your patient: an evidence-based practical guide. *PLoS One* 13:e0209205. 10.1371/journal.pone.0209205 30586435 PMC6306248

[B132] Sonnenborn U. (2016). *Escherichia coli* strain Nissle 1917-from bench to bedside and back: history of a special *Escherichia coli* strain with probiotic properties. *FEMS Microbiol. Lett.* 363:fnw212. 10.1093/femsle/fnw212 27619890

[B133] Sougioultzis S. Simeonidis S. Bhaskar K. Chen X. Anton P. Keates S. (2006). Saccharomyces boulardii produces a soluble anti-inflammatory factor that inhibits NF-kappaB-mediated IL-8 gene expression. *Natl. Library Med.* 343 69–76. 10.1016/j.bbrc.2006.02.080 16529714

[B134] Srinivasan R. Meyer R. Padmanabhan R. Britto J. (2006). Clinical safety of Lactobacillus casei shirota as a probiotic in critically ill children. *J. Pediatr. Gastroenterol. Nutr.* 42 171–173. 16456410 10.1097/01.mpg.0000189335.62397.cf

[B135] Stamps J. A.-M. (2012). *Drosophila* regulate yeast density and increase yeast community similarity in a natural substrate. *PLoS One* 7:e42238. 10.1371/journal.pone.0042238 22860093 PMC3409142

[B136] Sun Q. Liang X. Zheng Q. Gu W. Liu W. Xiao S. (2010). Resistance of *Helicobacter* pylori to antibiotics from 2000 to 2009 in Shanghai. *World J. Gastroenterol.* 16 5118–5121. 20976850 10.3748/wjg.v16.i40.5118 PMC2965290

[B137] Surawicz C. McFarland L. Greenberg R. Rubin M. Fekety R. Mulligan M. (2000). The search for a better treatment for recurrent Clostridium difficile disease: use of high-dose vancomycin combined with Saccharomyces boulardii. *Clin. Infect. Dis.* 31 1012–1017. 10.1086/318130 11049785

[B138] Surawicz C. M. Elmer G. W. Speelmen P. McFarland L. V. Chinn J. Van Belle G. (1989). Prevention of antibiotic-associated diarrhea by Saccharomyces boulardii: a prospective study. *Gastroenterology* 96 981–988. 10.1016/0016-5085(89)91613-2 2494098

[B139] Swansan H. (2015). Drug metabolism by the host and gut microbiota: a partnership. *Drug Metab. Dispos.* 43 1499–1504. 10.1124/dmd.115.065714 26261284 PMC4576677

[B140] Talwalkar A. Kailasapathy K. Peiris P. Arumungaswamy R. (2001). Application of RBGR - a simple way for screening of oxygen tolerance in probiotic bacteria. *Int. J. Food Microbiol.* 71 245–248. 10.1016/S0168-1605(01)00563-3 11789942

[B141] Tarr G. C.-A. (2019). Performance of stool-testing recommendations for acute gastroenteritis when used to identify children with 9 potential bacterial enteropathogens. *Clin. Infect. Dis. Official Publ. Infect. Dis. Soc. Am.* 69 1173–1182. 10.1093/cid/ciy1021 30517612 PMC7348586

[B142] Trinder M. D. (2017). Drosophila melanogaster as a high-throughput model for host-microbiota interactions. *Front. Microbiol.* 8:751. 10.3389/fmicb.2017.00751 28503170 PMC5408076

[B143] U.S. Food and Drug Administration (2014). *Types of Applications.* Silver Spring, MD: Food and Drug Administration.

[B144] U.S. Food and Drug Administration (2017). *The FDA’s Drug Review Process: Ensuring Drugs Are Safe and Effective.* Silver Spring, MD: Food and Drug Administration.

[B145] U.S. Food and Drug Administration (2019). *Questions and Answers on Dietary Supplements.* Silver Spring, MD: Food and Drug Administration.

[B146] U.S. Food and Drug Administration (2020). *New Dietary Ingredients in Dietary Supplements - Background for Industry.* Silver Spring, MD: Food and Drug Administration.

[B147] U.S. Food and Drug Administration; National Institute of Health, National Institute of Allergy and Infectious Diseases (2018). *Science and Regulation of Live Microbiome-based Products used to Prevent, Treat, and Cure Disease in Humans.* Available online at: https://www.fda.gov/media/128302/download (accessed May 7, 2021)

[B148] Valente G. Acurcio L. Freitas L. Nicoli J. Silva A. Souza M. (2019). Short communication: *In vitro* and *in vivo* probiotic potential of Lactobacillus plantarum B7 and Lactobacillus rhamnosus D1 isolated from Minas artisanal cheese. *J. Dairy Sci.* 102 5957–5961. 10.3168/jds.2018-15938 31128873

[B149] Vanderhoof J. A. Young R. (2008). Probiotics in the United States. *Clin. Infect. Dis.* 46(Suppl 2:S67-72) S144–S151. 10.1086/523339 18181726

[B150] Vecchio A. L. Guandalini S. Guarino A. (2015). Probiotics for prevention and treatment of diarrhea. *J. Clin. Gastroenterol.* 49 S37–S45. 10.1097/MCG.0000000000000349 26447963

[B151] Venugopalan V. Shriner K. A. Wong-Beringer A. (2010). Regulatory oversight and safety of probiotic use. *Emerg. Infect. Dis.* 16 1661–1665. 10.3201/eid1611.100574 21029521 PMC3294522

[B152] Villar-García J. G.-F. (2017). Impact of probiotic Saccharomyces boulardii on the gut microbiome composition in HIV-treated patients: a double-blind, randomised, placebo-controlled trial. *PLoS One* 12:e0173802. 10.1371/journal.pone.0173802 28388647 PMC5384743

[B153] Vitetta L. Saltzman E. T. Nikov T. Ibrahim I. Hall S. (2016). Modulating the gut micro-environment in the treatment of intestinal parasites. *J. Clin. Med.* 5:102. 10.3390/jcm5110102 27854317 PMC5126799

[B154] Wang L. Cao H. Liu L. Wang B. Walker W. A. Acra S. A. (2014). Activation of epidermal growth factor receptor mediates mucin production stimulated by p40, a lactobacillus rhamnosus GG-derived Protein. *J. Bio. Chem.* 289 20234–20244. 10.1074/jbc.M114.553800 24895124 PMC4106339

[B155] Wang T. D. (2020). Probiotics modulate intestinal motility and inflammation in Zebrafish models. *Zebrafish* 17 382–393. 10.1089/zeb.2020.1877 33232637

[B156] Wawrzyniak I. Poirier P. Viscogliosi E. Dionigia M. Texier C. Delbac F. (2013). Blastocystis, an unrecognized parasite: an overview of pathogenesis and diagnosis. *Therapeutic Adv. Infect. Dis.* 1 167–178. 10.1177/2049936113504754 25165551 PMC4040727

[B157] Whaling M. A. (2012). Perceptions about probiotic yogurt for health and nutrition in the context of HIV/AIDS in Mwanza, Tanzania. *J. Health Popul. Nutr.* 30 31–40. 10.3329/jhpn.v30i1.11273 22524117 PMC3312357

[B158] Xiao L. F. (2015). A catalog of the mouse gut metagenome. *Nat. Biotechnol.* 33 1103–1108. 10.1038/nbt.3353 26414350

[B159] Xiong T. Maheshwari A. Neu J. Ei-Saie A. Pammi M. (2020). An overview of systematic reviews of randomized-controlled trials for preventing necrotizing enterocolitis in preterm infants. *Neonatology* 117 46–56. 10.1159/000504371 31838477

[B160] Yason J. A. (2019). Interactions between a pathogenic Blastocystis subtype and gut microbiota: *in vitro* and *in vivo* studies. *Microbiome* 7:30. 10.1186/s40168-019-0644-3 30853028 PMC6410515

[B161] Yeung P. S. (2012). Species-specific identification of commercial probiotic strains. *J. Dairy Sci.* 85 1039–1051. 10.3168/jds.S0022-0302(02)74164-7 12086037

[B162] Yoo D. Kim I. Van Le T. Jung I. Yoo H. Kim D. (2014). Gut microbiota-mediated drug interactions between lovastatin and antibiotics. *Drug Metab. Dispos.* 42 1508–1513. 10.1124/dmd.114.058354 24947972

[B163] Yoon M. Y. Yoon S. S. (2018). Disruption of the gut ecosystem by antibiotics. *Yonsei Med. J.* 59 4–12. 29214770 10.3349/ymj.2018.59.1.4 PMC5725362

[B164] Young V. B. Dieterle M. G. Rao K. (2018). Novel therapies and preventative strategies for primary and recurrent Clostridium difficile infections. *Ann. N. Y. Acad. Sci.* 1435 110–138. 10.1111/nyas.13958 30238983 PMC6312459

[B165] Zbinden R. (1999). Inhibition of Saccharomyces boulardii (nom. inval.) on cell invasion of *Salmonella* typhimurium and Yersinia enterocolitica. *Micro Ecol. Health* 44 158–162. 10.1080/089106099435736

[B166] Zhang J. Zhang J. Wang R. (2018). Gut microbiota modulates drug pharmacokinetics. *Drug Metab. Rev.* 50 357–368. 10.1080/03602532.2018.1497647 30227749

[B167] Zhang Z. T. (2019). emystifying the manipulation of host immunity, metabolism, and extraintestinal tumors by the gut microbiome. *Signal Transduct Target Ther.* 4:41. 10.1038/s41392-019-0074-5 31637019 PMC6799818

[B168] Zollner-Schwetz I. Krause R. (2015). Therapy of acute gastroenteritis: role of antibiotics. *Clin. Microbiol. Infect.* 21 744–749. 10.1016/j.cmi.2015.03.002 25769427

